# Reply to Vrijdag et al. Comment on “Mankowska et al. Critical Flicker Fusion Frequency: A Narrative Review. *Medicina* 2021, *57*, 1096”

**DOI:** 10.3390/medicina58060765

**Published:** 2022-06-06

**Authors:** Natalia D. Mankowska, Anna B. Marcinkowska, Monika Waskow, Rita I. Sharma, Jacek Kot, Pawel J. Winklewski

**Affiliations:** 1 Applied Cognitive Neuroscience Lab, Department of Human Physiology, Medical University of Gdansk, 80-210 Gdansk, Poland; anna.marcinkowska@gumed.edu.pl; 2 2nd Department of Radiology, Medical University of Gdansk, 80-210 Gdansk, Poland; pawelwinklewski@wp.pl; 3 Institute of Health Sciences, Pomeranian University in Slupsk, 76-200 Slupsk, Poland; monika.waskow@apsl.edu.pl; 4 Department of Human Physiology, Medical University of Gdansk, 80-210 Gdansk, Poland; rita.sharma@gumed.edu.pl; 5 Department of Anaesthesiology and Intensive Care, Medical University of Gdansk, 80-210 Gdansk, Poland; 6 National Centre for Hyperbaric Medicine, Institute of Maritime and Tropical Medicine in Gdynia, Medical University of Gdansk, 80-210 Gdansk, Poland; jkot@gumed.edu.pl

Thank you very much for your interest and comments [1] on the review by Mankowska et al. [2], aiming at providing an overview of the use of critical flicker fusion frequency (CFFF) to investigate cognitive functions.

We agree with the authors of the Commentary [1] that the GABA_A_-receptor might be involved in nitrogen narcosis [3,4]. Yet, the precise molecular mechanisms of the adaptation of lipid bilayers to pressure are unknown and require further investigation [5]. The traditional view is that the lipid bilayer of the cellular membrane is the main target for anesthesia and pressure, while newer theories stress the role of transmembrane proteins. It is, however, likely that nitrogen may exert a pluripotent activity, targeting lipids and transmembrane proteins and implicitly affect water molecules at the lipid–solvent interface [5]. Consequently, the membrane theory and the GABA_A_ theory do not need to exclude each other [5,6]. Most importantly, presenting a discussion of the physiological mechanisms underlying anesthesiologic and pressure effects, although fascinating, was not the aim of the review. Rather, we strived to summarize the existing knowledge regarding the reliability of CFFF in the assessment of cognitive functioning versus other psychometric methods.

We have never implied that a reduction in CFFF while diving should be interpreted as a decline in cognitive performance solely due to nitrogen narcosis. On the contrary, we stressed that it is a multifactorial phenomenon and, particularly when diving below 50 msw (more than 608 kPa), there might be other variables such as oxygen toxicity. The dose–reaction relations between oxygen and cognitive functions is not clear and actually it is not known whether the increased excitability, and which forms of neuronal excitability, should be considered a part of the learning process or, rather, cellular manifestation of neuronal oxygen poisoning [7]. Consequently, it is not surprising that below 50 msw a further reduction in CFFF is not seen.

Indeed, “critical flicker fusion frequency” is not the same as “flickering light”. However, to the best of our knowledge, there are no studies yet that conclusively explain the mechanisms underlying the processing of flickering light, so we do not know how exactly decisions to perceive flicker or light continuity are made, and thus how the CFFF threshold is determined. We believe that it is impossible to understand CFFF without understanding these mechanisms, so describing CFFF in the context of flickering light was intended to suggest the need for further research using neuroimaging (e.g., electroencephalography), which could explain what dependencies and interactions we might expect when using the CFFF test. If we want to use the CFFF test as a measure of an individual’s arousal [8,9,10] or cognitive ability [11,12,13], including in pathological conditions such as epilepsy [14] or Alzheimer’s disease [15,16], we must understand how it interacts with the individual’s brain. In diving medicine, the use of electroencephalography to investigate the mechanisms underlying processes measured by CFFF seems particularly interesting in the light of the theory focused on the depth-related “effect on ligand-gated ion-channels in the postsynaptic membrane of excitable neurons”.
